# Bumblebee olfactory learning affected by task allocation but not by a trypanosome parasite

**DOI:** 10.1038/s41598-018-24007-9

**Published:** 2018-04-11

**Authors:** Callum D. Martin, Michelle T. Fountain, Mark J. F. Brown

**Affiliations:** 1 School of Biological Sciences, Royal Holloway University of London, Egham, Surrey United Kingdom; 2 NIAB EMR, East Malling Research, East Malling, Kent United Kingdom

## Abstract

Parasites can induce behavioural changes in their host organisms. Several parasite species are known to infect bumblebees, an important group of pollinators. Task allocation within bumblebee colonies can also cause differences in behaviour. Thus, task allocation may lead to context-dependent impacts of parasites on host behaviour. This study uses *Bombus terrestris* and its gut trypanosome *Crithidia bombi*, to investigate the effects of parasitism, task allocation (foraging or nest-work) and their interactions, on olfactory learning. Prior to undergoing the olfactory learning task, bees were orally infected with a field-realistic dose of *C. bombi*, and observed to determine task allocation. Parasitism did not significantly affect olfactory learning, but task allocation did, with foragers being significantly more likely to learn than nest bees. There was no significant interaction between parasitism and task. These results suggest that *C. bombi* is unlikely to affect pollination services via changes in olfactory learning of its host if bees are under no environmental or nutritional stress. However, wild and commercial colonies are likely to face such stressors. Future studies in the field are needed to extrapolate our results to real world effects.

## Introduction

Parasites are highly prevalent in ecosystems with approximately 50% of all animal species thought to be parasitic^1,2^. One way that parasites can impact hosts is through behavioural alteration^3–5^. Such parasite-induced behavioural changes may be manipulative and enhance the fitness or transmission of the parasite, but they can also be non-manipulative, benefitting host rather than parasite fitness^5^. Understanding such manipulations is increasingly important, as parasites have also been implicated in population declines of numerous taxa^6–9^. Bumblebees are one such group; they host a wide variety of parasite species, and parasitic infections are thought to be one of the key drivers of their declines in Europe and The Americas^6,10–14^.

A common parasite in many bumblebee species is the gut trypanosome *Crithidia bombi* (Lipa and Triggiani, 1980). This parasite is often found at prevalences of 10–30% in bumblebee populations, and has been recorded at prevalences as high as 80% at specific sites^15–19^. *C. bombi* has also been introduced to South America via its host *Bombus terrestris*. Here, the parasite has spread rapidly, and is one of several potential causes for the decline of the native *Bombus dahlbomii*^6^. *C. bombi* has been shown to effect host behaviour in several ways^19–22^. Experiments using artificial flowers have found motor-learning rate, learning based on colour cues, and foraging rate to be reduced in infected bumblebees^20–22^. In addition, previous work observed a correlation between parasitism and a lower likelihood of pollen collection in the field^19^.

While the mechanism behind these changes is unknown, one potential explanation is that parasites activate the immune system of their hosts, which can subsequently interact with the nervous system^23–25^. *C. bombi* is known to activate the bumblebee immune system^26–29^, and both bumblebees and honeybees have been shown to display impaired olfactory learning when their immune systems are artificially activated^24,25^. Thus, *C. bombi* infection may alter host behavior via activation of the immune system.

Context is key for understanding parasite impacts on host behaviour, and as *C. bombi* displays context-dependent virulence, with virulence increasing during periods of food stress or during energetically demanding stages of the host’s life cycle^30,31^, behavioural impacts may also be modified by host context. Task allocation within bumblebee colonies provides differing contexts, with individual workers more regularly performing either energetically demanding foraging tasks outside the nest, or less energetically demanding tasks based inside the nest (e.g. brood care)^32–34^. Furthermore, foraging activity has been shown to reduce immunocompetence in bumblebees^35^. The differing energy demands associated with performing these tasks, and their trade-offs with immunocompetence, could be sufficient for context-dependent behavioural impacts to be induced by the parasite.

This study investigated the effect of *C. bombi* infection, task allocation, and their interactions on the olfactory learning of the common European bumblebee species *Bombus terrestris* L. (1758). We hypothesised that *C. bombi* would alter olfactory learning ability, potentially via interactions between the immune and nervous systems. We further predicted that this alteration would be dependent on task allocation within the bumblebee colony. Olfactory learning was assessed using the proboscis extension reflex (PER) experimental paradigm, a classical conditioning procedure^36^. During PER experimentation, bees undergo a series of trials where they can learn to associate an odour (conditioned stimulus) with a sugar solution reward (unconditioned stimulus)^36^. During each trial whether or not a bee displayed a conditioned response was recorded.

## Results

150 bees from 4 colonies were deemed motivated enough to undergo PER trials, with 80 of these being from the control treatment and 70 from the parasite treatment. 39 of the 150 were judged to be unmotivated (26 control bees and 13 parasite bees) during the trials after showing 3 consecutive non-responses to the sucrose solution. These bees were not included in further analyses. Of the 111 motivated bees, 57 were from the parasite treatment group and 54 from the control group. 55 of the 111 (49.5%) bees showed at least one conditioned response. No bees responded to the control trials.

**Figure 1:**
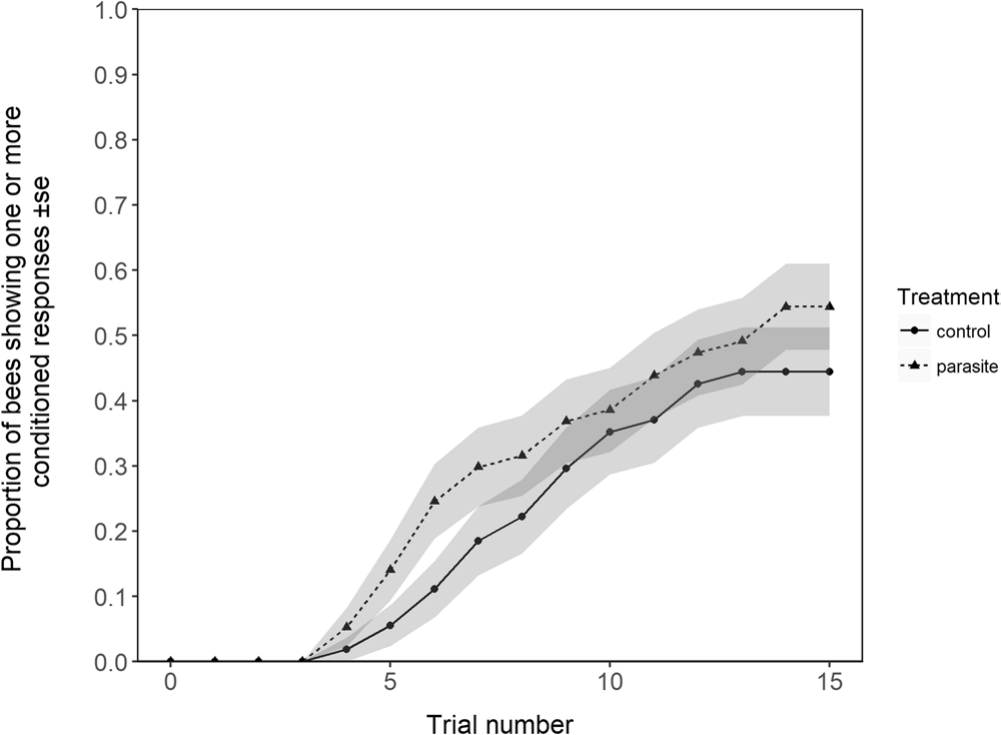
Cumulative proportion of parasitised and control bees to have shown at least one conditioned response throughout the duration of the trials. Grey shaded area around lines represents ± the standard error of the mean.

While a greater percentage of parasitized bees (54.4%) showed a conditioned response than control bees (44.4%) (Fig. 1), treatment was not a significant covariate in the Cox regression analysis (Cox proportional hazards model: Hazard ratio (*HR*) = 1.13, *P* = 0.79). Similarly, although parasitised bees displayed, on average, a greater number of conditioned responses during the full duration of the trials, this was not significant (GLMM: *z* = 0.69, *P* = 0.49).

Infection intensity was also not a significant explanatory variable for either the likelihood of a bee showing one or more conditioned responses (Cox proportional hazards model: *HR* = 1.10, *P* = 0.25), or for the total number of conditioned responses a bee displayed (GLMM: *z* = 1.88, *P* = 0.06).

**Figure 2:**
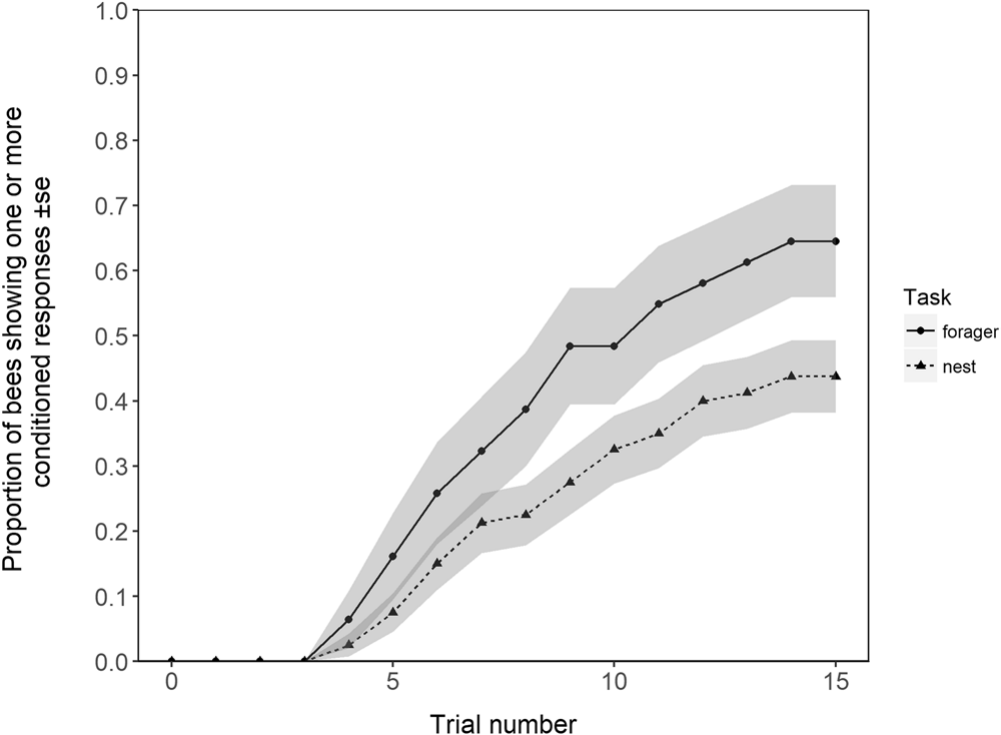
Cumulative proportion of forager and nest bees to have shown at least one conditioned response throughout the duration of the trials. Grey shaded area around lines represents ± the standard error of the mean.

Of the 111 motivated bees, 31 were categorised as foragers, while the remaining 80 bees were categorised as nest bees. 64.5% of forager bees showed at least one conditioned response, whereas only 43.8% of the nest bees did (Fig. 2), and the task of the bee (forager or nest) was a significant predictor of learning in the Cox regression model (Cox proportional hazards model: *HR* = 2.50, *P* = 0.029).

Forager bees also displayed, on average, a greater number of conditioned responses than nest bees throughout the PER trials, but this was not a significant difference (GLMM: *z* = 1.55, *P* = 0.12).

**Figure 3:**
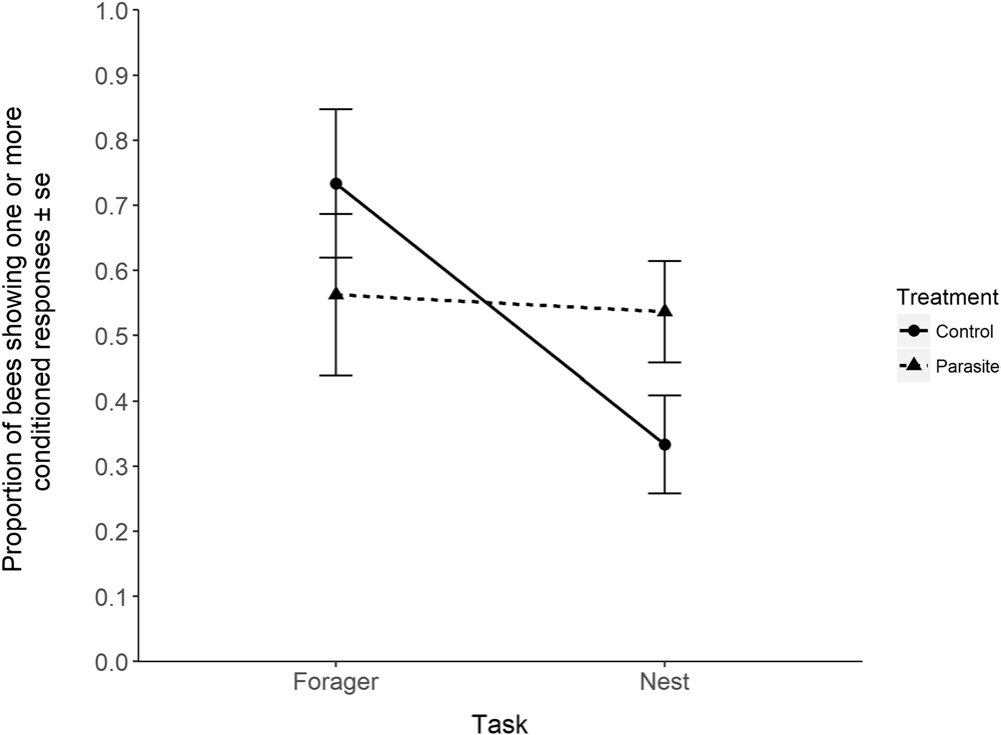
Visualisation of the non-significant interaction (see results for statistics) between treatment and task, with proportion of bees that showed one or more conditioned responses as the response variable. Error bars represent ± standard error of the mean.

The difference in the likelihood of a bee showing one or more conditioned responses was greater between the foragers and nest bees in the control group than in the parasitised group (Fig. 3). In the control group 73.3% (11 out of 15) of the foragers showed at least one conditioned response, compared to 33.3% (13 out of 39) of the nest bees. In the parasitised group 56.3% (9 out of 16) of foragers and 53.7% (22 out of 41) of nest bees showed one or more conditioned responses. However, the hypothesised interaction between the treatment and the task that the bee performs (foraging or nest tasks) was not a significant explanatory variable in the Cox model output (Cox proportional hazards model: *HR* = 0.43, *P* = 0.14).

The interaction term between treatment and task was also not a significant explanatory variable (GLMM: *z* = −0.98, *P* = 0.33) of the total number of conditioned responses that bees displayed. Here, the control and parasitised bees showed a very similar relationship for foragers and nest bees.

## Discussion

In our experiment, infection with the parasite *C. bombi* had no significant effect on the olfactory learning ability of *B. terrestris*. However, the task the individual was allocated within the colony did affect the likelihood of the bee showing at least one conditioned response, with forager bees more likely to learn than nest bees. Interactions between task allocation and treatment were non-significant, contrasting with our initial hypothesis that task-dependent parasite-induced learning alterations could occur in this system.

It is surprising that we do not observe a significant effect in the parasite treatment group given that *C. bombi* is known to activate the immune system of *B. terrestris*^26^, and that activated bumblebee and honeybee immune systems can interact with the nervous system to cause impairments in cognitive function^24,25^. However, in bumblebees, reduced learning ability as measured by PER methodologies was only observed in bees that were starved of pollen^25^, and pollen is crucial for proper functioning of the immune system^37^. During our experiment, bees had an *ad libitum* supply of pollen directly into the nest, so they were not in a state of nutritional stress. This could explain why parasitic infection had no effect on olfactory learning in this experiment, since bees may have had sufficient nutrition to support both functioning immune systems and other physiological functions. However, it should be noted that *C. bombi* has been shown to impair learning ability in non-nutritionally stressed bees^20–22^, although these studies did not use PER methodologies. Further work with nutritionally stressed bees is needed to clarify this relationship.

We found the infection intensity of *C. bombi* to have no significant effect on learning ability. This is contrary to other experiments that have found increasing *C. bombi* infection intensity to negatively impact learning ability^20,21^. These experiments did, however, use different experimental set-ups to test learning (i.e. not PER) and were performed on a different bumblebee species (*Bombus impatiens*), which could have contributed to the differing results. Furthermore, the number of cells in the *C. bombi* inoculum and the infection intensities observed in Gegear *et al*.^21^, one of the previously cited experiments, were much higher than in our experiment. This may further explain why, in contrast to the Gegear *et al*.^21^ study, no effect of infection intensity was observed in our experiment.

In this study foragers were more likely to show one or more conditioned responses than nest bees. Foraging is a complex task, requiring the individual to differentiate between the quality and quantity of a wide variety of potential forage resources. Given this, one might expect foragers to have increased cognitive function, and indeed in both ants and honeybees, individuals that forage have been shown to perform better at learning tasks than individuals based in the nest^38,39^. Similar patterns have been observed in *B. terrestris* colonies when the queen is present, however, in contrast to our experiment, these patterns were no longer observed upon removal of the queen^40^. In the bumblebee *Bombus occidentalis*, bees with more foraging experience were found to be better learners, and the foraging activity caused an increase in the size of the mushroom bodies, an area of the brain associated with learning and memory^41,42^. It is also possible that differential gene expression between task allocated bees could enhance the learning ability of foragers^43^. The results presented here on *B. terrestris* add further evidence to this pattern, strongly suggesting that task allocation can alter learning ability in social insects, and at least across bumblebee species.

The superior learning ability of foragers could alternatively be explained by the size of the individual. Bumblebees that more regularly perform foraging tasks are generally larger than their nest based counterparts^44^, and larger body size has been associated with increased learning ability^41,45^. However, in our experiment foragers did not have a larger body size, and body size was not a significant predictor of learning ability in any of the models.

Interaction terms between treatment and task were not significant predictors of either of the response variables analysed, suggesting that task-dependent parasite-induced learning alterations do not occur in this host-parasite system, or that we lacked sufficient power to detect such an effect. However, this result should be interpreted with caution as in our experimental set up bees could only forage in the flight arena (100 × 75 × 50 cm), whereas in the wild foragers may travel hundreds or even thousands of metres in a single foraging bout^46–49^. This means that the energy demands of foraging in this experiment are greatly reduced compared to those of wild foragers, which subsequently could decrease the likelihood of observing any context dependent effects, as any trade-off between energy consumption for flight and for immune system upregulation is much less severe.

Further differences exist between the foraging arena and the wild environment in the prior conditioning that bees experience. In the foraging arena, it is necessary to ensure that bees have not associated any odour with a reward prior to the PER protocol, but in the wild, foragers will have made associations between particular flower odours and the rewards that they can provide. Foraging from nectar feeders in the arena does, however, still require that bees use visual cues and learning in order to locate food, which does replicate the behaviour of wild foragers, albeit at a lower level of complexity.

It is possible that our observation and definition of forager and nest bees, led to our results being conservative, and the difference in learning ability between foragers and nest bees could in fact be greater. Our observation protocol allowed us to be sure that the animals we labelled foragers had indeed foraged, but it is possible that a proportion of the bees that were judged to be nest bees left the nest and foraged at a time when there was no ongoing observation of the flight arenas. If this did occur, these individuals would have been included in the nest bee category, but they may have displayed the enhanced learning ability of a forager, which could dampen the effect of task allocation on learning that is being observed.

The results presented here provide evidence that *C. bombi* has no meaningful effect on *B. terrestris* olfactory learning, and that the task that the bee performs is a more important factor in predicting learning ability. It can be concluded that *C. bombi* is unlikely to affect pollination services via changes in olfactory learning of its host, at least in an environment where food is abundant, but it could still impair pollination services through previously described impacts on mortality and motor learning^20,22,30,31^. This experiment does not support the existence of task-dependent parasite-induced learning alterations, however their presence cannot be ruled out given the restricted foraging environment that bees were constrained to. Future research should focus on investigating cognitive function of parasitised bees in stressful conditions, or in a more field realistic situation where foraging is more energetically demanding and where food supply is not *ad libitum*.

## Methodology

### Queen collection

Wild *B. terrestris* queens were collected from Windsor Great Park, Surrey, UK (Latitude: 51.417677, longitude: −0.604263). Queens were collected between the 11^th^ March and 7^th^ April 2015. Collected bees had their faeces screened under a microscope at x400 magnification for the presence of *C. bombi*. Those individuals that did harbour the parasite (n = 32) were placed into individual plastic nest boxes (W = 6.7, L = 12.7, D = 5 cm) and provisioned with ad libitum pollen and nectar. When the first workers began to emerge, the colonies were transferred to larger plastic nest boxes (W = 22.5, L = 29, D = 13 cm) where they were kept for the remainder of the experiment. These colonies did not forage outside of their colony box at any point during their life cycle, and they were not subjected to a regular light cycle. These colonies were then used as a source of parasitic cells for the inoculation of commercial colonies during experiments. The infectiveness of a parasite to its host can vary between different host populations^50,51^. Thus, having several infected wild bees meant that there was a variety of different parasite strains, which increased the likelihood that strains were present that could successfully infect commercial colonies. Furthermore, we used multiple strains to maximise the chances of observing broad impacts of the parasite in bumblebees, rather than strain specific effects.

### Commercial colonies

**Figure 4:**
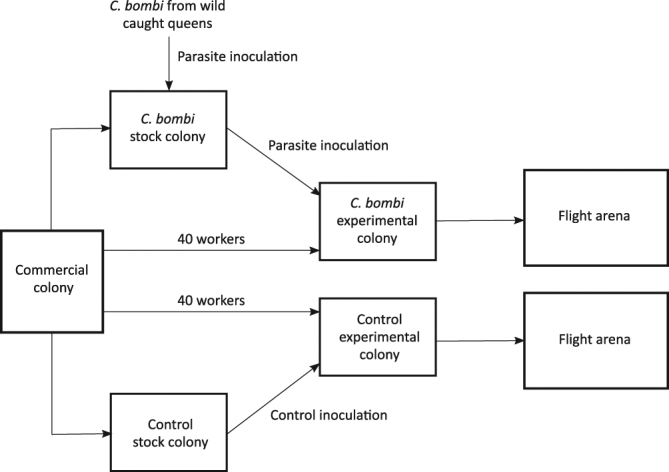
Overview of the creation of ‘experimental’ and ‘stock’ colonies from a single commercial colony. The same process was repeated for 4 commercial colonies.

Four commercial colonies were imported from Biobest (between November 2015 and January 2016) to be used for experimentation. Upon arrival 15 workers were removed from each colony and their faeces screened for the presence of parasites^52^. All four colonies were uninfected and thus kept for the experiment. The queen was removed from each commercial colony, then each colony was split into four sub-colonies. Two of these sub-colonies consisted of 40 workers and half of the original brood each, these were the ‘experimental’ colonies and they were assigned a treatment (control or parasite) and placed into wooden nest boxes (W = 14, L = 24, D = 10 cm). The remaining two sub-colonies were the ‘stock’ colonies. These were made up by splitting the bees remaining from the original commercial colonies after the set-up of the ‘experimental’ colonies. One of these sub-colonies was inoculated with *C. bombi* collected from faeces of bees from all of the wild colonies. The other colony was left uninfected as control stock. These stock colonies were used for any subsequent inoculations of their corresponding experimental colony (see Fig. 4 for overview of colony splitting process). This process allowed for filtration of all the *C. bombi* strains, so that only those strains infective to a particular commercial colony were used for subsequent infections.

In total, we had 8 experimental sub-colonies, 4 with the parasite treatment and 4 with control treatment, and we also had 8 stock sub-colonies, each one corresponding to an experimental sub-colony. This split colony design helped account for the large intercolony variation in learning ability that exists^53^.

The wooden nest boxes containing the experimental sub-colonies were then connected to flight arenas (W = 75, L = 100, D = 50 cm) via a gated tunnel. Gravity feeders filled with 40% sugar solution were placed within the arenas to allow the bees to forage, and *ad libitum* pollen was provided to each colony directly into the nest box. The stock colonies were placed into plastic nest boxes and stored in a dark room with *ad libitum* pollen and nectar.

### Crithidia bombi purification and inoculation

Inocula were made by taking a minimum of 10 bees from the parasite or control stock colonies. The faeces of these bees were collected and then purified following the method used by Baron *et al*.^54^ modified from Cole^55^. The faeces were diluted with 0.9% Ringer’s solution to make 1 ml of total solution (dilution 1). The solution was centrifuged at 0.8 G for two minutes, the supernatant was then removed and placed into another centrifuge tube (dilution 2), whilst the remaining pellet was diluted and re-suspended with another 1 ml of Ringer’s solution. This process was repeated until 8 dilutions had been prepared. Dilutions 4, 5, and 6 were taken and centrifuged at 8 G for 1 minute, the supernatant removed, and the pellets mixed with 100 µl of Ringer’s solution. A small amount of the resulting solution was placed in a Neubauer chamber, allowing for the *C. bombi* cells to be counted and the concentration of the parasite in the solution to be calculated. The amount of solution that contained 10,000 parasite cells was calculated and this dose was diluted with 40% sugar solution to make a 20 µl solution which was fed to individual bees. The same protocol was followed to make a control inoculum using the faeces of bees from the control stock colonies. Any bee that did not consume the inoculum was not used for further experimentation.

### Callow marking

Experimental sub-colonies that had been connected to flight arenas were observed every day for the emergence of callow workers. Workers were individually marked with uniquely numbered Opalith tags on the day they emerged, so the age of each marked bee was known. Marked bees were inoculated between the ages of 3 to 5 days using the method previously described. Bees were then left to harbour the parasite for a further 7 to 10 days post-inoculation. This time period was chosen as it has previously been shown that the parasite load 7 to 10 days post-inoculation is relatively high and remains stable^56^. During the bee marking process, the flight arenas were observed in both the morning and afternoon. Bees could forage in flight arenas at all times, and any marked bee that was observed foraging on a nectar feeder was judged to be a forager, whilst bees never observed to forage were judged to be nest bees.

### Olfactory learning

Olfactory learning was assessed using the proboscis extension reflex (PER) experimental paradigm, where bees learn to associate an odour (conditioned stimulus) with a sugar solution reward (unconditioned stimulus)^36^. This method has been used for over 50 years to test learning and memory in honeybees (*Apis mellifera*) with great success^57^, and has more recently been used successfully on bumblebees^41,58^.

Between 13:00 and 15:00 on the afternoon before the PER experiment, marked workers 6–9 days post-inoculation (7–10 days on the following day of experimentation) were taken from the nest box and flight arena, placed on ice for approximately 5 minutes until quiescent, and then harnessed. The harness prevented the bee from flying and crawling, but allowed the bee to move its head (see supplementary material Figure S1 for photo of harnessed bee). All workers were fed to satiety with 40% sugar solution 2 hours after harnessing, and were left upright in a container overnight.

The following morning between 08:00–09:00, bee responsiveness was tested by touching their antennae with a droplet of nectar solution. Those bees responding with a proboscis extension were deemed to be sufficiently motivated to be used for behavioural assays, and were fed a small droplet of nectar solution to maintain motivation 15 minutes before the experiment.

The PER experiment itself was carried out between 09:00–12:30. During the experiment each harnessed bee was individually placed in an odour extraction hood. Air flow into the hood was controlled by a programmable logic controller computer. The air flow was directed onto the bee via an odour tube placed 3 cm away from the bee. A piece of filter paper soaked in 4 µl of lemon scented oil was placed inside the odour tube, and this filter paper was replaced every 20 trials to keep the intensity of the odour constant. The bees were exposed to 15 seconds of air flow in total, the first 5 seconds being clean air and the final 10 seconds being the odour. The reward was presented to the bee 6 seconds into the odour stimulus by touching its antenna with a 0.8 µl droplet of 40% nectar solution using a Gilmont syringe. If presentation of the reward elicited a proboscis extension response then the bee was fed the nectar droplet, but if the bee did not respond to the reward then it did not receive any nectar. A conditioned response occurred when the bee extended its proboscis on exposure to the odour stimulus without needing presentation of the nectar solution on its antennae. In this case, the bee was fed the nectar droplet. Each bee underwent 15 odour exposures with a 12-minute interval between each exposure.

Three control trials were interspersed randomly within the final 10 odour exposures. During a control trial an unscented airflow was directed onto the bee for the duration of the trial, and no reward presentation occurred. These control trials were performed to check that bees were not becoming conditioned to the airflow rather than the scent. Any individual that appeared to be conditioned to the airflow, i.e., showed a conditioned response during a control trial, was removed from the analysis. Bees were also deemed unmotivated and excluded from the analysis if they did not respond to the nectar stimulus for 3 consecutive trials.

After completing the PER trials, bees were placed into a freezer at −20 °C. The thorax width of each bee was recorded as a measure of bee body size, which in some cases has been shown to effect learning ability^45,59^.

### Parasite infection intensity

The parasite intensity was quantified for all bees from the parasite treatment following a similar methodology to Baer and Schmid-Hempel^60^. This was done by combining the hind-gut of a bee with 100 µl of 0.9% ringers solution. The mixture was then ground-up in a 500 µl reaction tube and mixed in a vortex mixer for 5 seconds. A *C. bombi* cell count was performed on 0.02 µl of the gut solution using a Neubauer haemocytometer.

### Data Analysis

All statistical analyses were carried out using ‘R’ programming software^61^. The total number of conditioned responses was analysed using a negative binomial mixed effects model in the ‘glmmADMB’ package^62,63^. Mixed effects Cox proportional hazards models, in the package ‘coxme’^64,65^, were used to analyse the learning rate and final proportion of bees that displayed one or more conditioned responses. Mixed modelling techniques were used to allow for the colony each bee came from to be accounted for as a random effect. Co-variables used in the models included ‘treatment’, ‘task (forager or nest)’, ‘thorax width’, ‘age’, ‘infection intensity’, and the interaction term between ‘treatment’ and ‘task’. These variables were chosen as we had *a priori* reasons to believe that they could affect learning or interact with the treatment to effect learning. There was no correlation between variables and models were not overdispersed. Validation of the Cox model was carried out to check it was not violating the proportional hazards assumption. Mixed effects models were validated by visual inspection of plots of the model residuals plotted against the fitted values.

### Data availability statement

The dataset generated and analysed during the current study is available from the corresponding author upon request. It is also available on Figshare (https://figshare.com/s/82b35fadd236c053915b).

## Electronic supplementary material

Supplementary material
