# Supplementary material for: Bumblebee olfactory learning affected by task allocation but not by a trypanosome parasite
Source: Sci Rep. 2018 Apr 11;8:5809. doi: 10.1038/s41598-018-24007-9 (PMC5895637; doi:10.1038/s41598-018-24007-9)
Supplement: Supplementary file 1 — Supplementary material [file 41598_2018_24007_MOESM1_ESM.pdf]

Bumblebee olfactory learning affected by task allocation but not by a trypanosome parasite

Callum D. Martin, Michelle T. Fountain & Mark J. F. Brown

Supplementary material

Harnessed bumblebee

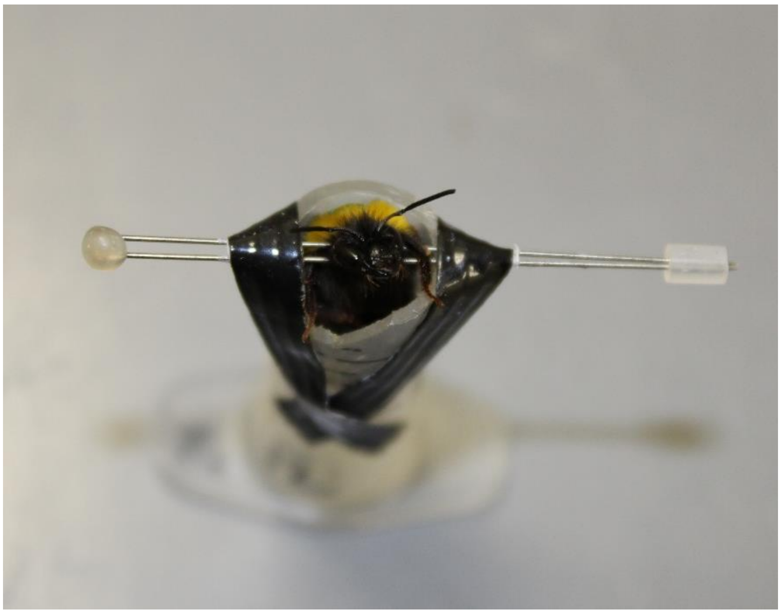

Figure S1. *Harnessed bee before PER trials*
